# Immune Infiltration and Clinical Outcome of Super-Enhancer-Associated lncRNAs in Stomach Adenocarcinoma

**DOI:** 10.3389/fonc.2022.780493

**Published:** 2022-03-03

**Authors:** Li Peng, Jiang-Yun Peng, Dian-Kui Cai, Yun-Tan Qiu, Qiu-Sheng Lan, Jie Luo, Bing Yang, Hai-Tao Xie, Ze-Peng Du, Xiao-Qing Yuan, Yue Liu, Dong Yin

**Affiliations:** ^1^ Guangdong Provincial Key Laboratory of Malignant Tumor Epigenetics and Gene Regulation, Guangdong-Hong Kong Joint Laboratory for RNA Medicine, Sun Yat-Sen Memorial Hospital, Sun Yat-Sen University, Guangzhou, China; ^2^ Medical Research Center, Sun Yat-Sen Memorial Hospital, Sun Yat-Sen University, Guangzhou, China; ^3^ Department of Hepatobiliary Surgery, Sun Yat-sen Memorial Hospital, Sun Yat-sen University, Guangzhou, China; ^4^ Department of Gastrointestinal Surgery, Sun Yat-sen Memorial Hospital, Sun Yat-sen University, Guangzhou, China; ^5^ Department of Clinical Laboratory, The First Affiliated Hospital, Hengyang Medical School, University of South China, Hengyang, China; ^6^ Central Laboratory, Department of Pathology, Shantou Central Hospital, Shantou, China; ^7^ Breast Tumor Center, Sun Yat-sen Memorial Hospital, Sun Yat-sen University, Guangzhou, China; ^8^ Institute of Digestive Disease of Guangzhou Medical University, The Sixth Affiliated Hospital of Guangzhou Medical University, Qingyuan People’s Hospital, Qingyuan, China

**Keywords:** super-enhancer (SE), long non-coding RNA (IncRNA), stomach adenocarcinoma (STAD), immune infiltration, clinical outcome, TM4SF1-AS1, T cell, PD1

## Abstract

Super-enhancers (SEs) comprise large clusters of enhancers that highly enhance gene expression. Long non-coding RNAs (lncRNAs) tend to be dysregulated in cases of stomach adenocarcinoma (STAD) and are vital for balancing tumor immunity. However, whether SE-associated lncRNAs play a role in the immune infiltration of STAD remains unknown. In the present study, we identified SE-associated lncRNAs in the H3K27ac ChIP-seq datasets from 11 tumor tissues and two cell lines. We found that the significantly dysregulated SE-associated lncRNAs were strongly correlated with immune cell infiltration through the application of six algorithms (ImmuncellAI, CIBERSORT, EPIC, quantiSeq, TIMER, and xCELL), as well as immunomodulators and chemokines. We found that the expression of SE-associated lncRNA TM4SF1-AS1 was negatively correlated with the proportion of CD8+ T cells present in STAD. TM4SF1-AS1 suppresses T cell-mediated immune killing function and predicts immune response to anti-PD1 therapy. ChIP-seq, Hi-C and luciferase assay results verified that TM4SF1-AS1 was regulated by its super-enhancer. RNA-seq data showed that TM4SF1-AS1 is involved in immune and cancer-related processes or pathways. In conclusion, SE-associated lncRNAs are involved in the tumor immune microenvironment and act as indicators of clinical outcomes in STAD. This study highlights the importance of SE-associated lncRNAs in the immune regulation of STAD.

## Introduction

Stomach cancer is the fifth most common cause of cancer-related morbidity and fourth most common cause of mortality worldwide, accounting for over 1,000,000 new cases and approximately 769,000 deaths in 2020 (1). Infection with *Helicobacter pylori*, smoking, and diets high in nitrate and nitrite can lead to stomach cancer (2). With progress in diagnosis and treatment, the quality of life of patients with stomach cancer can be improved. Stomach adenocarcinoma (STAD), aka gastric adenocarcinoma, is the most common histological type of stomach cancer (approximately 95%) (3), and patients still have a poor prognosis (2, 4). Biomarkers such as PD1 and PDL1 are used increasingly often as immunotherapy for STAD, which benefits patients (2). Therefore, identifying credible biomarkers is important for the early identification of STAD and novel molecular targeting treatments.

Super-enhancers (SEs) comprise large clusters of activated enhancers in close proximity to one another that maintain cell-type-specific identity and fate (5). These enhancers are generally within 12.5 kb in genomic distance (6). Considerable progress regarding research on SE-associated protein-coding genes, such as HOXB8 (7), MEIS1 (8) and MYC (9) has been made recently. In addition, SEs reportedly drive the expression of long noncoding RNA (lncRNA) in various cancers, except stomach cancer. Importantly, Wen et al. reported a genome-wide reprogramming of enhancer and SE landscape in cases of STAD, which lead to dysregulated local and regional cancer gene expression (10). This finding indicates that SEs are vital for the occurrence and development of multiple cancers that involve STAD that regulate the expression of tumor-related genes. However, the roles of SE-associated long non-coding RNAs in STAD remain unclear.

## Materials and Methods

### STAD Cell Lines

The STAD cell lines AGS and MKN45 were cultured in F12K and DMEM containing 10-15% fetal bovine serum at 37°C in a humidified atmosphere containing 5% CO_2_.

### Chromatin Immunoprecipitation Sequencing

The AGS cells were cross-linked with formaldehyde and sonicated using a Bioruptor (Diagenode, Belgium), and the precipitate was removed *via* centrifugation. Protein A/G magnetic beads (Pierce, USA) were incubated overnight with a histone-3-lysine-27 acetylation (H3K27ac) antibody (Abcam, USA) at 4°C. The DNA fragments were eluted and recycled for chromatin immunoprecipitation sequencing (ChIP-seq) on a BGISEQ-500 platform (BGI-Shenzhen, China).

### ChIP-Seq Analysis

Raw ChIP-seq data of MKN45 cells and 11 STAD tumors were downloaded from the Gene Expression Omnibus database. The downloaded raw data and our sequencing data were ordered *via* trimming, Bowtie2 read mapping, filtering, and MACS2 peak calling. Bam files were converted into bigwig files using bamCoverage. Hockey stick plots and positional information of super-enhancers were analyzed using the rank ordering of super enhancers algorithm (5). The Integrative Genomics Viewer was used to visualize the bigwig files of the ChIP-seq data.

### Survival Analysis

The STAD dataset was downloaded from the Cancer Genome Atlas (TCGA; https://cancergenome.nih.gov/), which includes 375 tumor samples and 32 non-malignant stomach tissue samples. Patients with STAD who were included in the TCGA were divided into high and low expression groups according to the median. GraphPad software was used to draw all survival-related graphs. Statistical significance was set at *p*<0.05.

### Correlation of lncRNAs With the Tumor Immune Microenvironment and Immune Markers

Immune cell abundance was evaluated using six algorithms, namely ImmuCellAI (11), CIBERSORT (12), EPIC (13), quantiSeq (14), TIMER (15), and xCELL (16). The correlation of the lncRNA expression matrix with immune cell abundance was analyzed.

Immune markers including immunomodulators (immunoinhibitors and immunostimulators), MHC molecules, chemokines and chemokine receptor were downloaded from TISIDB (http://cis.hku.hk/TISIDB/download.php). The expression of these markers was from TCGA database. The correlation of the lncRNA expression matrix with immune markers level was also analyzed in STAD. 

### siRNA Transfection

The TM4SF1-AS1 small interference RNA (siRNA) sequences were designed and synthesized by GenePharma (Suzhou, China). The siRNA was diluted using sterile DEPC water and transfected into AGS and MKN45 cells using Lipofectamine RNAiMAX Transfection Reagent (Invitrogen, USA). The siRNA sequences of TM4SF1-AS1 are listed in **Table 1**.

**Table 1:** SiRNA sequences.

Gene name	siRNA sequences	
	Sense	Antisense
siTM4SF1-AS1#1	GGCAUUGACUGUGCAACUCCU	GGAGGAGUUGCACAGUCAAUG
siTM4SF1-AS1#2	GCCCUGGUUGAGGCUUUGAAA	UCUUUCAAAGCCUCAACCAGG
siTM4SF1-AS1#3	GGUGAAACUCUCCACUCCUTT	AGGAGUGGAGAGUUUCACCTT
siTM4SF1-AS1#4	GUUCAGACCAGUGAGAUUUTT	AAAUCUCACUGGUCUGAACTT
siNC	UUCUCCGAACGUGUCACGUTT	ACGUGACACGUUCGGAGAATT

### RNA Isolation and qRT-PCR

RNA was isolated from cells using the TRIzol method. Cells in a 6-well plate were lysed with 1 mL Trizol in each well, and 200 μL chloroform was added, shaken, mixed, and centrifuged at 12,000 rpm at 4°C for 10 min after being incubated for 5 min. Next, 500 μL isopropanol was added, mixed, and centrifuged. After being washed twice with 75% alcohol, the RNA precipitate was dissolved in diethyl pyrocarbonate water. RNA was reverse transcribed to first-strand cDNA using Hifair III 1st Strand cDNA Synthesis SuperMix for qPCR (gDNA digester plus) (Yeasen, China).

ChamQ Universal SYBR qPCR Master Mix (Vazyme, China) was used to perform qRT-PCR on a CFX96 Real-Time System (Bio-Rad, USA). Relative mRNA expression was calculated using the 2^−ΔΔCT method and normalized to that of GAPDH. The primers are shown in **Table 2**.

**Table 2:** Primer sequences.

Gene name	Primer sequences	
	Forward	Reverse
TM4SF1-AS1	CCCATGGATTTGAGAAGGCTG	AAGCAGGAGTGGAGAGTTTCA
GAPDH	CTGGGCTACACTGAGCACC	AAGTGGTCGTTGAGGGCAATG
E1	TTACGCGTGCTAGCCCGGGCTCGAGGGGAAATTCCTCTCACTTCAAT	TGAGATGCAGATCGCAGATCTCTTTCAACTAAAAGTGAACT
E2	TTACGCGTGCTAGCCCGGGCTCGAGAATATGCATTACAAAAAACTTTCCA	TGAGATGCAGATCGCAGATCACCAGAAGGGCACACTT
E3	gene synthesis	
E4	TTACGCGTGCTAGCCCGGGCTCGAGACATGGCCTTTTGACCTTAT	TGAGATGCAGATCGCAGATTAGGGTCATGCTTATTTGGA
E5	gene synthesis	
NC	TTACGCGTGCTAGCCCGGGCTCGAGTATATAAAGAATATTTTGATACC	TGAGATGCAGATCGCAGATACAGACATTTGTATATATTCAC

### CD8+ T Cell Separation From Human PBMCs

CD8+ T cells were separated from human PBMCs using the EasySep™ Human CD8+ T Cell Isolation Kit (STEMCELL Technologies, Canada). Samples were prepared at the indicated cell concentrations within the volume range. The isolation cocktail was added to the required tube, mixed, and incubated. Vortex RapidSpheres™, add it to the sample and mix them. The recommended medium was added to top up the sample to the indicated volume, then mixed by gently pipetting the sample up and down 2–3 times. The tube was placed into the magnet without a lid and incubated. The magnet was then picked up and the magnet and tube were inverted in one continuous motion, pouring the enriched cell suspension into a new tube.

### T Cell Activation and *In Vitro* Killing Assays

First, stomach cancer cells were stained using the CellTrace CFSE Cell Proliferation Kit (Invitrogen, USA) for 20 min at 37°C. Activated CD8+ T cells (2 × 10^4^) were generated by incubation with ImmunoCult™ Human CD3/CD28 T Cell Activator (STEMCELL Technologies, Canada) at 37°C and 5% CO_2_ for up to 3 days, and were then added to cancer cells. Both activated CD8+ T cells and cancer cells were collected to be stained with propidium iodide (3.75 mM solution, 1:500 final dilution). These cells were harvested and analyzed using flow cytometry after 12 h (Beckman CytoFLEX, USA).

### Hi-C Analysis

Hi-C data of two STAD samples, T2000877 (GSM3333325) and T990275 (GSM3356360), were downloaded from the Gene Expression Omnibus database (GSE118391; https://www.ncbi.nlm.nih.gov/geo/query/acc.cgi?acc=GSE118391). The results of this analysis were visualized and graphed using WashU tool (http://epigenomegateway.wustl.edu/browser/).

### Vector Construction

Each of the five enhancer regions (E1–E5) and their negative control (NC) were amplified using PCR or gene synthesis, then cloned into the pGL3-promoter vector. The primers used for PCR amplification of each enhancer are listed in **Table 2**.

### Dual Luciferase Reporter Gene Assay

The vectors were transfected into STAD AGS and MKN45 cells, and pRL-TK plasmids were co-transfected as a normalization control. Luciferase assays were conducted using the Dual-Luciferase Reporter Assay System (Promega, USA).

### RNA-Seq

Total RNA was extracted from cells using the TRIzol method as previously described, then qualified and quantified using a Nano Drop and Agilent 2100 bioanalyzer (ThermoFisher Scientific, USA). The mRNA library was constructed and sequenced on a BGIseq500 system (BGI-Shenzhen, China).

### Gene Set Enrichment Analysis (GSEA)

GSEA 4.1.0 software (http://www.broadinstitute.org/gsea/index.jsp) was downloaded and employed to analyze critical gene enrichment pathways between different groups based on FPKM values, which reflect relative gene expression.

### Statistical Analysis

All data are presented as mean ± SD. The Student’s t-test or analysis of variance was performed using either SPSS or GraphPad 8.0.1 software. Values were considered statistically significant at *p*<0.05 (*, *p*<0.05; **, *p*<0.01; ***, *p*<0.001).

## Results

### Identification of Super-Enhancer-Associated lncRNAs in STAD Samples and Cells

**Figure 1:**
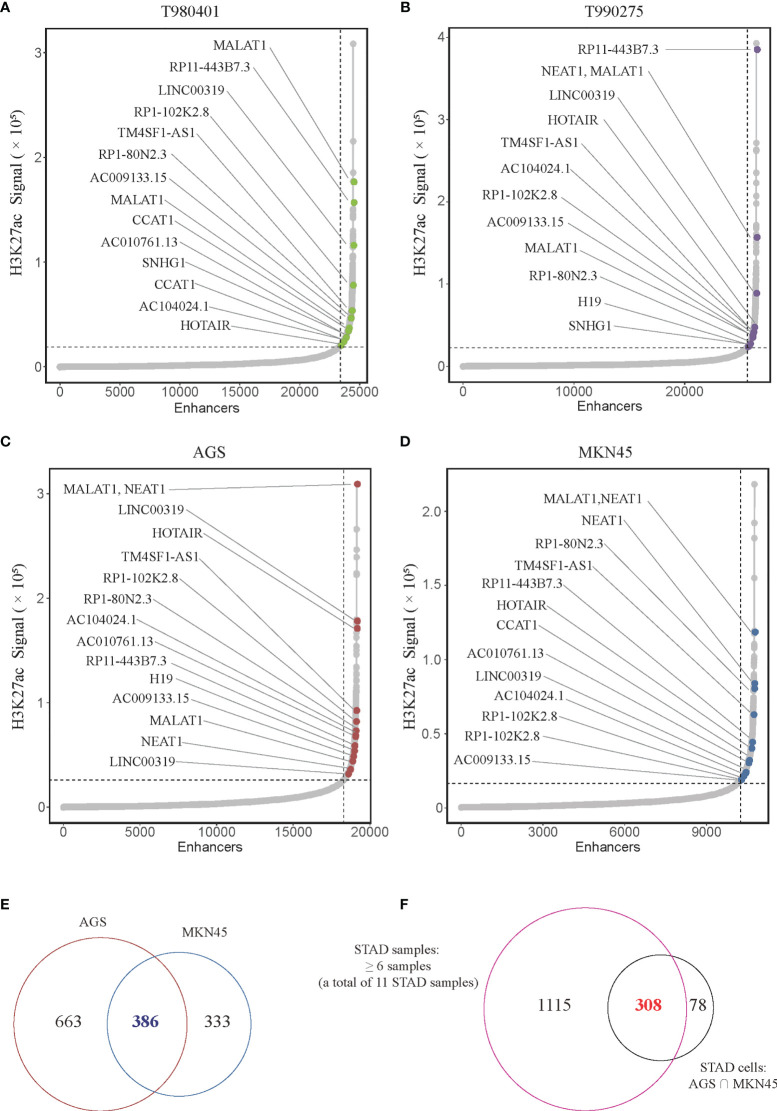
Super-enhancer-associated lncRNAs present in STAD samples and cells. **(A–D)** Hockey stick plot of SE-associated lncRNAs in two tumor samples (T980401 and T990275) and two STAD cells (AGS and MKN45). Some representations of novel and known SE-associated lncRNAs are shown in color on the curve. **(E)** Venn diagram showing the intersection of SE-associated lncRNAs in two STAD cells (AGS and MKN45). **(F)** Venn diagram displaying the intersection of the SE-associated lncRNAs shared in two STAD cells and the SE-associated lncRNAs in at least 6 of 11 STAD samples. ChIP-seq with the H3K27ac antibody in AGS cell lines was from this study, and other H3K27ac ChIP-seq in 11 STAD tissue samples and MKN45 cell lines were downloaded from GEO database (GSE117953; https://www.ncbi.nlm.nih.gov/geo/query/acc.cgi?acc=GSE117953).

LncRNA plays a crucial role in biological processes and are regulated by SEs in several types of cancer. However, no studies on the relationship between lncRNAs and super-enhancers in STAD have been conducted to date. We first performed ChIP-seq with the H3K27ac antibody in AGS cell lines to identify SE-associated lncRNA in STAD, and downloaded raw data of 11 tissue samples and MKN45 cell lines from GEO database (GSE117953). We identified 1722, 1273, 1514, 2150, 2017, 1434, 1536, 1935, 983, 1978, and 2393 SE-associated lncRNAs in 11 STAD samples, respectively (T980401, T990275, and other 9 samples), and 1049 and 719 in two STAD cells, respectively (AGS and MKN45). Hockey stick plots of two representative tumor samples and both cells showed some SE-associated lncRNA (**Figures 1A–D**). Unsurprisingly, some common and vital lncRNAs that promote tumor progression are at the forefront of the H3K27ac signal, such as MALAT1, H19, and CCAT1 (**Figures 1A–D**). We also identified some novel SE-associated lncRNA in STAD (**Figures 1A–D**). According to the results of ChIP-seq, SE-associated lncRNAs in AGS and MKN45 cell lines intersected, and 386 common SE-associated lncRNAs were obtained (**Figure 1E**). The 386 SE-associated lncRNAs of the above two cell lines and the SE-associated lncRNAs obtained in the same way, which were present in over six STAD tissue samples, were intersected and 308 common SE-associated lncRNAs were obtained (**Figure 1F**). We analyzed 308 SE-associated lncRNAs in STAD samples and cells using ChIP-seq data against the H3K27ac antibody.

### Expression Profile and Clinical Outcome of Super-Enhancer-Associated lncRNAs in STAD Patients

**Figure 2:**
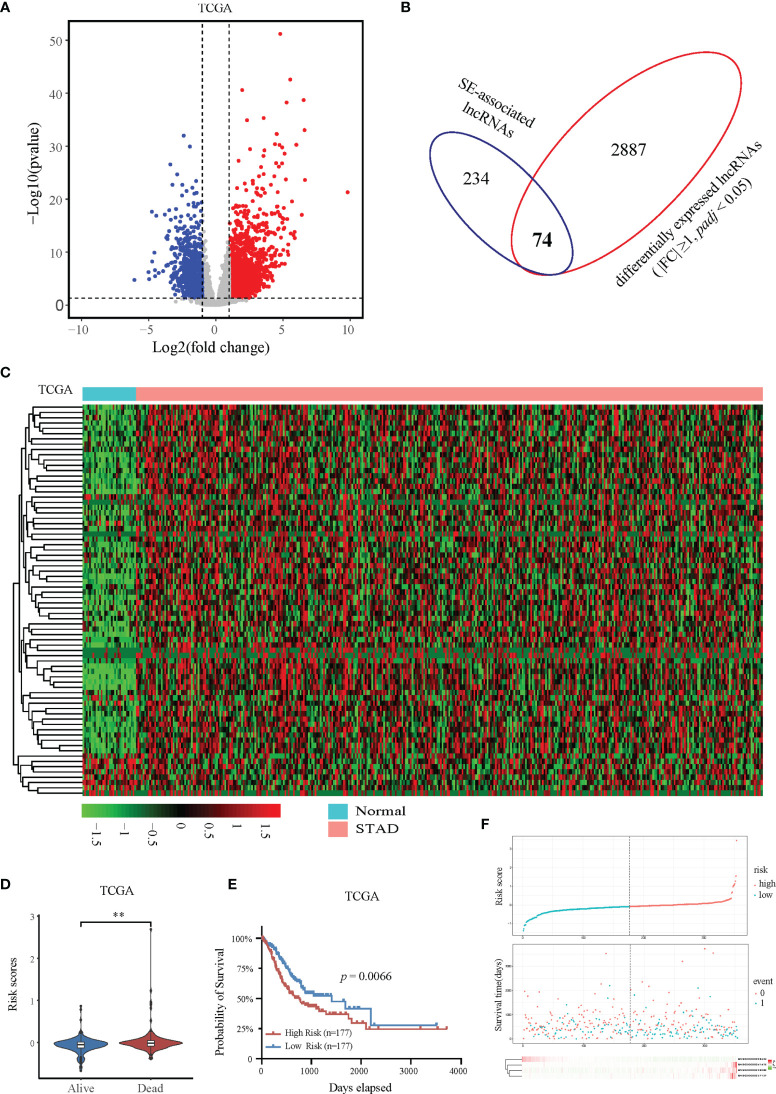
Expression and prognostic analysis of super-enhancer-associated lncRNAs in patients with STAD. **(A)** Volcano plot showing the expression changes of SE-associated lncRNAs in STAD samples compared with normal stomach tissues. The red dots represent up-regulated lncRNAs (FC ≥ 1, Q value < 0.05), and the blue dots represent down-regulated lncRNAs in STAD patients (FC ≤ -1, Q value < 0.05). **(B)** Venn diagram representing the intersection of 2961 differentially expressed lncRNAs in patients with STAD and the 308 SE-associated lncRNAs identified above. **(C)** Expression profiling of 74 differentially expressed SE-associated lncRNAs in human STAD samples and normal stomach tissues. **(D)** The risk scores of alive or dead STAD patients predicted by expression of SE-associated lncRNAs from TCGA dataset. **(E)** The prognosis of SE-associated lncRNAs in STAD patients with high risk and low risk based on the RiskScore signature. **(F)** The distributions of the four lncRNAs in groups between high and low risk in STAD patients. The top was the risk score of STAD patients calculated according to the RiskScore signature and split into two groups on basis of medium score. The middle represented the survival status. The bottom heatmap showed the differential profile of four lncRNAs. The expression and clinical data of all lncRNAs were downloaded from TCGA database (https://www.cancer.gov/). **Indicates *p* < 0.01.

To further determine the expression profile and clinical value of SE-associated lncRNA, we first analyzed the expression changes of all lncRNAs in patients with STAD from TCGA database. The expression of 2961 lncRNAs in STAD patients were significantly upregulated or downregulated compared with that of normal samples (**Figure 2A**). The 2961 lncRNAs with altered expression in patients with intersected with the 308 SE-associated lncRNAs from the previous results, and 74 SE-associated and differentially expressed lncRNAs were obtained in total (**Figure 2B**). The heatmap revealed overall differential expression of these 74 SE-associated lncRNAs in normal stomach and STAD samples (**Figure 2C**). We next utilized the least absolute shrinkage and selection operator (LASSO) algorithm to evaluate the prognosis role of 74 SE-associated lncRNAs in STAD patients. So that four SE-associated lncRNAs were included in this prognosis model according to the minimum criteria. We first observed the risk scores in alive and dead STAD patients, and found that the risk scores in dead STAD patients was indeed higher than those in alive patients (**Figure 2D**). We divided these SE-associated lncRNAs into two groups, high risk and low risk on basis of the RiskScore signature. High risk group was showed to be correlated with worse prognosis in STAD patients compared with low risk group (**Figure 2E**). The distribution of risk scores in STAD patients were showed (**Figure 2F**). Taken together, we elicited 74 differentially expressed SE-associated lncRNAs and assessed their correlation with the clinical importance of STAD patients.

### The SE-Associated lncRNAs Are Correlated With Immune Cell Infiltration in STAD

**Figure 3:**
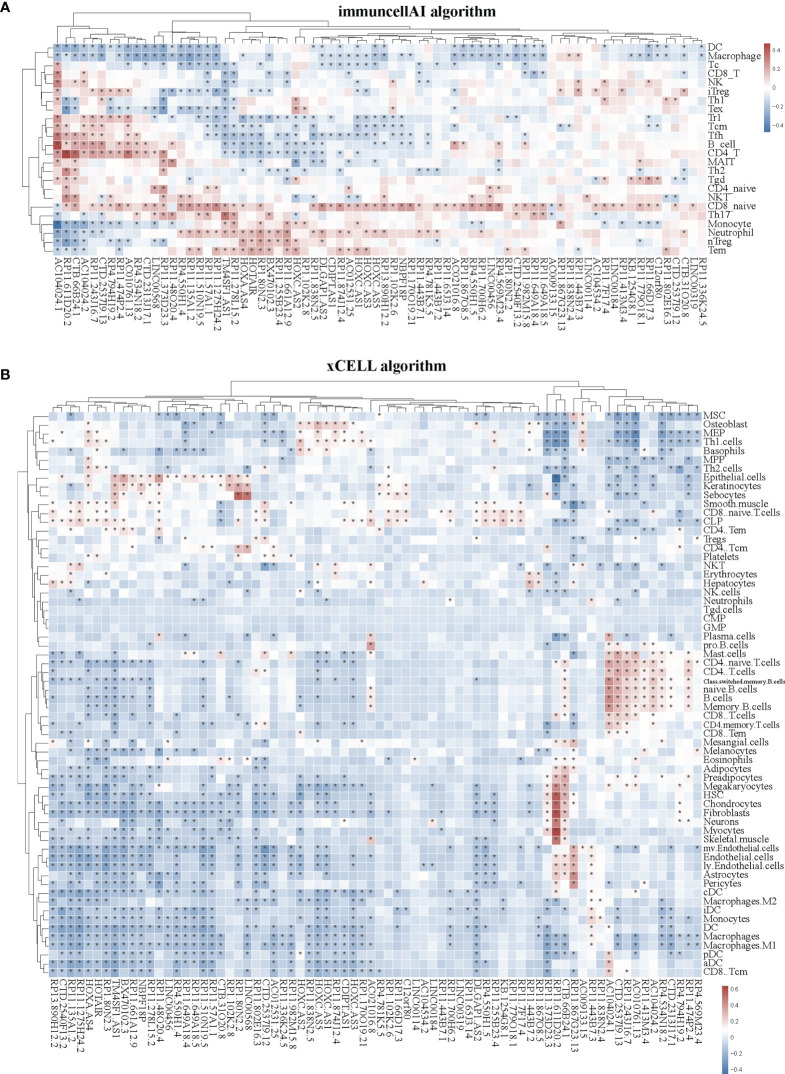
Correlation between the expression of super-enhancer-associated lncRNA and immune cell infiltration in patients with STAD. **(A)** Cluster analysis of the correlation between 74 SE-associated lncRNAs expression and immune cells abundance calculated by using the ImmuCellAI algorithm. **(B)** Cluster analysis of the correlation between 74 SE-associated lncRNAs and immune cells abundance calculated with the Xcell algorithm. Red indicates positive correlation, while blue indicates negative correlation. The asterisk (*) signifies a significant correlation *p* < 0.05. The expression of 74 SE-associated lncRNAs were downloaded from TCGA database (https://www.cancer.gov/). Immune cell infiltration was calculated by using the ImmuCellAI (http://bioinfo.life.hust.edu.cn/ImmuCellAI/#!/) and Xcell algorithm (https://xcell.ucsf.edu/) in STAD.

Immune cells always affect the occurrence and development of tumors in the tumor microenvironment, and the relationship between immune cells and tumors can be used to develop specific drugs to inhibit tumor progression, such as PD1 and PDL1 inhibitors. We then examined whether SE-associated lncRNAs in STAD are related to the infiltration of immune cells. We used six algorithms to analyze the correlation between SE-associated lncRNAs and immune cells infiltration, namely ImmuCellAI, CIBERSORT, EPIC, quantiSeq, TIMER, and Xcell, respectively. It was found that all 74 differentially expressed SE-associated lncRNAs in STAD were significantly correlated with the immune cell infiltration using the ImmuCellAI (**Figure 3A**) and Xcell algorithms (**Figure 3B**). These immune cells included T and B cells, DC, neutrophils, and macrophage cells. In addition, most differentially expressed SE-associated lncRNAs in STAD were found to be significantly correlated with the immune cell infiltration by the other four algorithms, namely CIBERSORT (**Supplementary Figure S1A**), EPIC (**Supplementary Figure S1B**), quantiSeq (**Supplementary Figure S1C**), and TIMER (**Supplementary Figure S1D**). These data indicated that a correlation exists between differentially expressed SE-associated lncRNAs and immune cell infiltration in STAD.

### SE-Associated lncRNAs Are Correlated With Immune Markers in STAD

**Figure 4:**
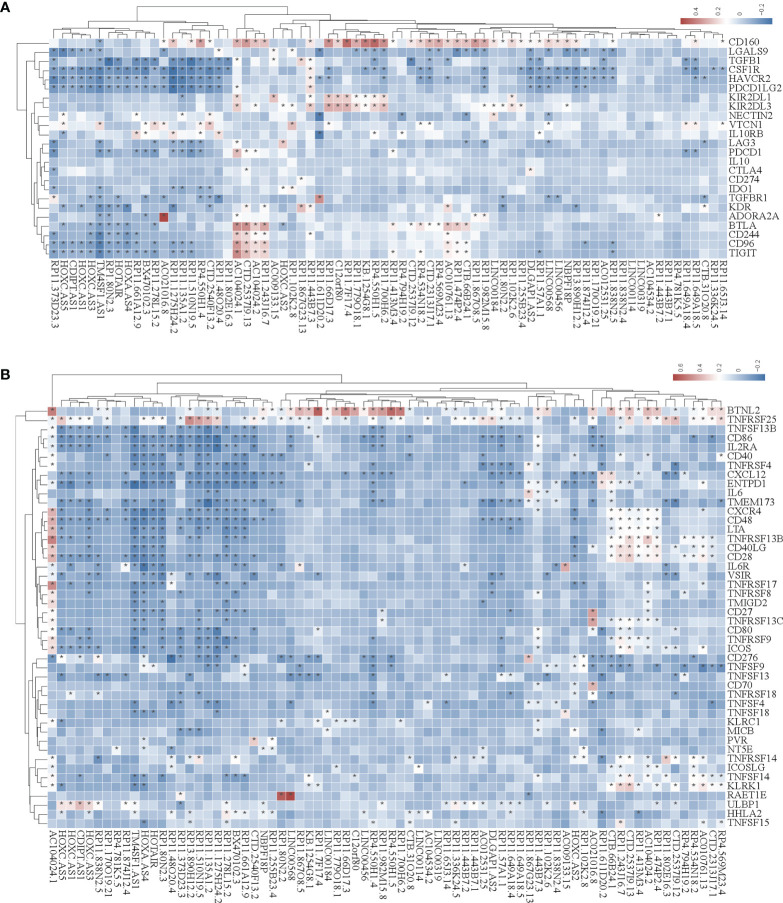
The correlation between the expression of super-enhancer-associated lncRNA and immunomodulators in STAD. **(A)** Cluster analysis of the expression correlation between 74 SE-associated lncRNAs and immunoinhibitor-related markers. **(B)** Cluster analysis of the expression correlation between 74 SE-associated lncRNA strands and Immunostimulator-related markers. Red indicates positive correlation, while blue signifies negative correlation. The asterisk (*) indicates significant correlation (*p* < 0.05). The list of immunomodulators including immunoinhibitory and immunostimulator was downloaded from TISIDB database (http://cis.hku.hk/TISIDB/download.php). The expression of 74 SE-associated lncRNAs and immunomodulators were downloaded from TCGA database (https://www.cancer.gov/).

**Figure 5:**
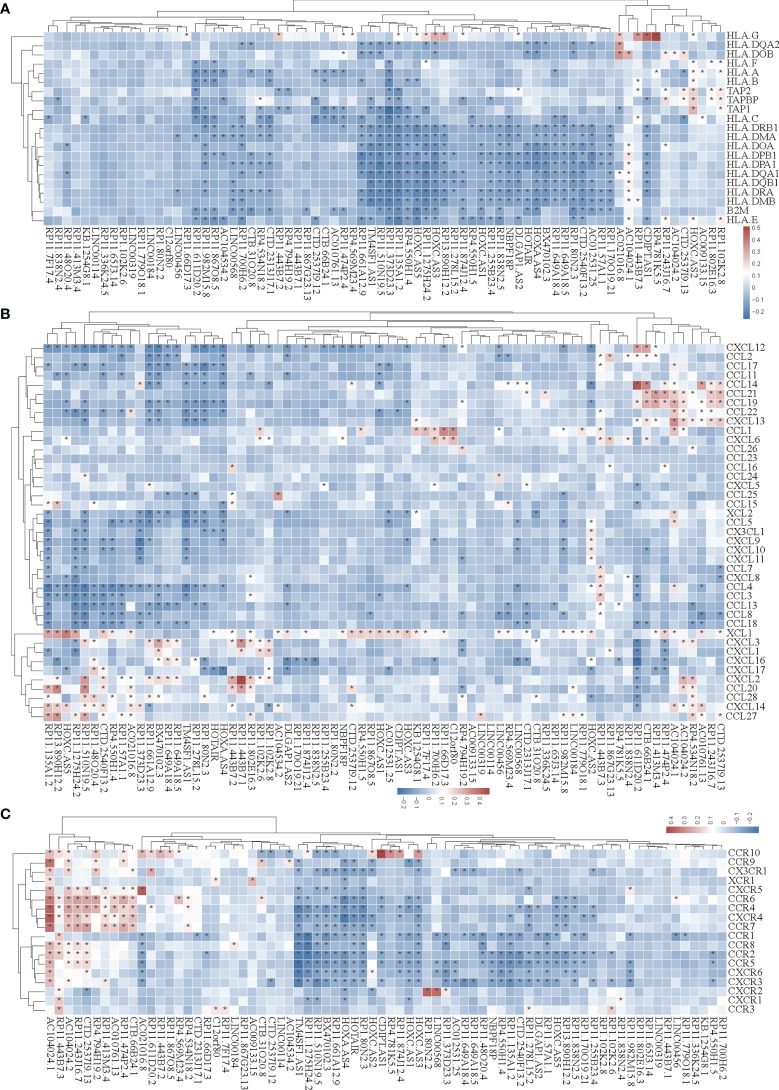
Correlation analysis of the expression of super-enhancer-associated lncRNA and chemokines in STAD cells. **(A)** Results of cluster analysis of the expression correlation between 74 SE-associated lncRNAs and MHC molecules. **(B)** Cluster analysis results of the expression correlation between 74 SE-associated lncRNA and chemokines. **(C)** Results of the cluster analysis of the expression correlation between 74 SE-associated lncRNAs and chemokine receptors. Red signifies a positive correlation, while blue indicates negative correlation. The asterisk (*) is used to signify significant correlation (*p* < 0.05). The list of MHC molecules, chemokines and chemokine receptors was downloaded from TISIDB database (http://cis.hku.hk/TISIDB/download.php). The expression of 74 SE-associated lncRNAs, MHC molecules, chemokines and chemokine receptors were downloaded from TCGA database (https://www.cancer.gov/).

In the tumor immune microenvironment, immune-related genes play a vital role in the regulation of tumor killing, including immunomodulators and chemokines. Therefore, we investigated whether SE-associated lncRNAs in STAD are related to immune makers. The list of immunomodulators and chemokines was downloaded from TISIDB database (http://cis.hku.hk/TISIDB/download.php). We first analyzed the correlation between SE-associated lncRNAs and immunomodulators in STAD and found that both immunoinhibitors and immunostimulators exhibited a significant correlation with SE-associated lncRNAs (**Figures 4A, B** **)**. In addition, SE-associated lncRNAs were significantly correlated with MHC molecules (**Figure 5A**).

We then analyzed the correlation between SE-associated lncRNAs and chemokines in STAD and found that chemokines and chemokine receptors were significantly related to SE-associated lncRNAs (**Figures 5B, C** **)**. In summary, these data show that SE-associated lncRNAs in STAD are related to immunomodulators and chemokines, and they participate in tumor immune regulation of tumors.

### TM4SF1-AS1 Is Involved in T Cell Mediated Immunity, and Predicts Immune Response to Anti-PD1 Therapy

**Figure 6:**
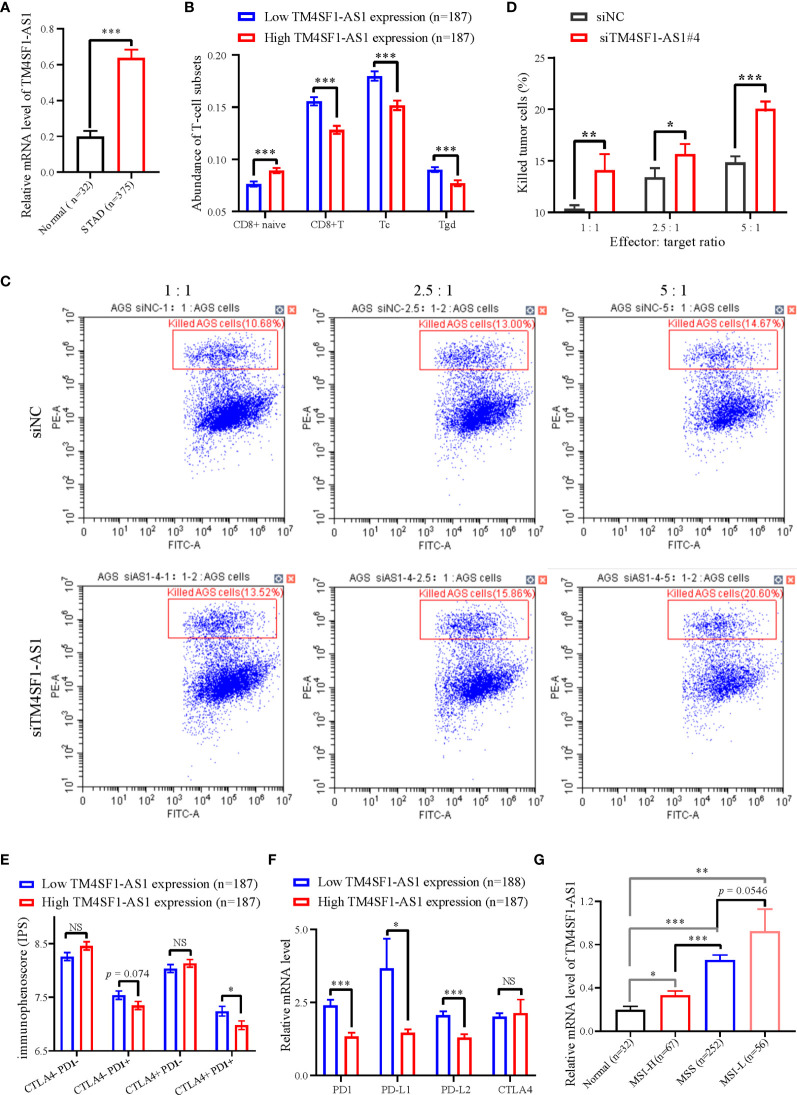
The expression of TM4SF1-AS1 and its role in the tumor immune microenvironment in STAD. **(A)** Expression changes of TM4SF1-AS1 in human STAD samples compared with normal samples. **(B)** The abundance of CD8+ T cell between high and low expression of TM4SF1-AS1. **(B, C)** Flow cytometry showing the effect of TM4SF1-AS1 knockdown on the killing activities of activated T cells in AGS cells. **(C)** Immunophenoscore of CTLA- PD1-, CTLA- PD1+, CTLA+ PD1- and CTLA+ PD1+ patients in the groups with high and low TM4SF1-AS1 expression, respectively. **(D)** Expression of PD1, PD-L1, PD-L2 and CTLA4 in the high and low TM4SF1-AS1 expression groups. **(E)** Expression of TM4SF1-AS1 in groups among normal tissues, MSI-H, MSS and MSI-L. **(F)** Expression of PD1, PD-L1, PD-L2 and CTLA4 in the high and low TM4SF1-AS1 expression groups. **(G)** Expression of TM4SF1-AS1 in groups among normal tissues, MSI-H, MSS and MSI-L. The expression of TM4SF1-AS1, PD1, PD-L1, PD-L2 and CTLA4 were downloaded from TCGA database (https://www.cancer.gov/). Immunophenoscore information of CTLA- PD1-, CTLA- PD1+, CTLA+ PD1- and CTLA+ PD1+ patients were downloaded from TICA database (https://tcia.at/home). NS stands for “no significance”, * refers to *p* < 0.05, ** indicates *p* < 0.01, and *** signifies *p* < 0.001.

We selected a CD8+ T cell-related lncRNA, TM4SF1-AS1, as a representation to investigate the role of SE-associated lncRNAs in tumor immune regulation of STAD. First, the expression of TM4SF1-AS1 in STAD samples was analyzed, and the expression of TM4SF1-AS1 was found to be significantly upregulated in STAD samples, which differed from normal stomach samples (**Figure 6A**). We then observed the abundant differences in TM4SF1-AS1 expression in CD8+ T cells and classified subtypes as either high or low TM4SF1-AS1 expression. The abundance of CD8 naïve cells in the group with high TM4SF1-AS1 expression was found to be significantly lower than that of samples with low TM4SF1-AS1 expression. The abundance of CD8^+^ T, Tc, and Tgd cells was the opposite (**Figure 6B**).

We also performed an *in vitro* T cell killing assay to observe the killing effect of activated T cells on tumor cells with TM4SF1-AS1 interference. First, we found that the #3 and #4 siRNA against TM4SF1-AS1 had the best knockdown efficiency (**Supplementary Figures S2A, B**), and we selected siRNA#4 for further experimentation. Subsequently, the T cell killing assay showed that TM4SF1-AS1 interference significantly weakened the killing effect of activated CD8+ T cells on tumor cells in a T cell concentration-dependent manner (**Figures 6C, D** **)**.

Furthermore, we analyzed immunophenoscore in CTLA- PD1-, CTLA- PD1+, CTLA+ PD1-and CTLA+ PD1+ patients between the two groups with high and low TM4SF1-AS1 expression, respectively. Immunophenoscore information of CTLA- PD1-, CTLA- PD1+, CTLA+ PD1- and CTLA+ PD1+ patients were downloaded from TICA database (https://tcia.at/home). The immunophenoscore of CTLA^-^ PD1+ and CTLA+ PD1+ samples in the group with high TM4SF1-AS1 expression was significantly lower than that of samples with low TM4SF1-AS1 expression, while no significant difference was observed in CTLA- PD1- and CTLA+ PD1- patients between the two groups (**Figure 6E**). We analyzed the expression of PD1, PD-L1, PD-L2, and CTLA4 in both groups. The expression of PD1, PD-L1, and PD-L2 was significantly higher in the group with high TM4SF1-AS1 expression (**Figure 6F**). and the expression of CTLA4 did not differ significantly between the high and low TM4SF1-AS1 expression groups (**Figure 6G**). In summary, these results indicate that TM4SF1-AS1 is involved in the T cell-mediated killing effect of tumor cells and monitors the immune response to anti-PD1 therapy.

### TM4SF1-AS1 Is Driven by SEs in STAD

**Figure 7:**
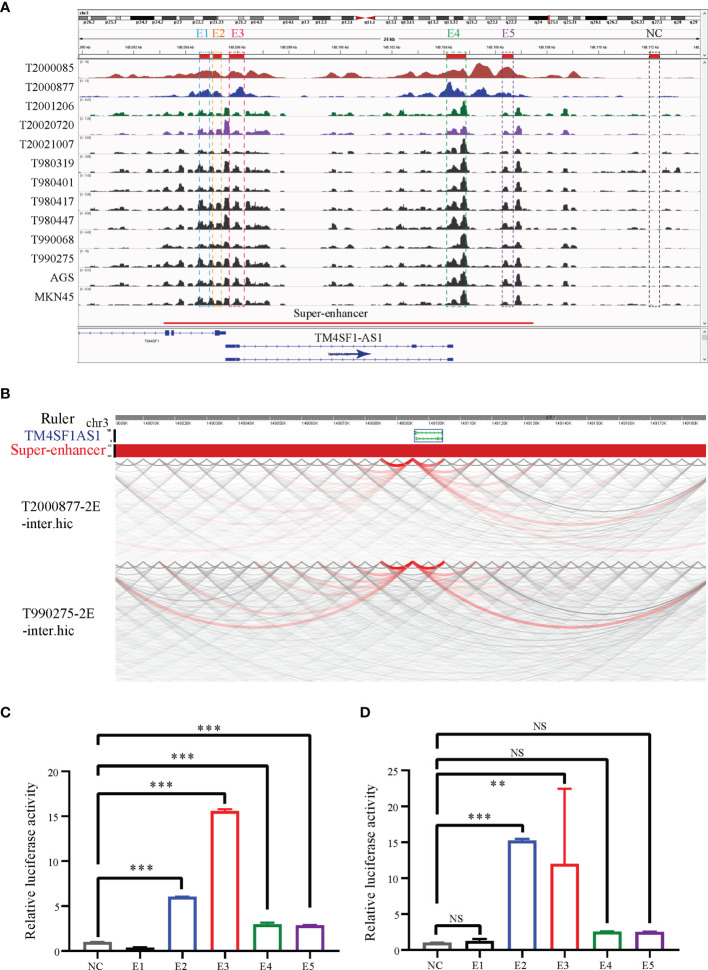
TM4SF1-AS1 is driven by the super-enhancer in STAD. **(A)** ChIP-seq profiles of H3K27ac in 11 STAD samples and 2 STAD cells (AGS and MKN45). The super-enhancer region is divided into five enhancers, and their location information are showed. ChIP-seq with the H3K27ac antibody in AGS cell lines was from this study, and other H3K27ac ChIP-seq in 11 STAD tissue samples and MKN45 cell lines were downloaded from GEO database (GSE117953; https://www.ncbi.nlm.nih.gov/geo/query/acc.cgi?acc=GSE117953). **(B)** Hi-C data analysis of super-enhancer regions and promoter of TM4SF1-AS1 in two tumor samples (T980401 and T990275). Hi-C data in two tumor samples (T980401 and T990275) was downloaded from GEO database (GSE118391; https://www.ncbi.nlm.nih.gov/geo/query/acc.cgi?acc=GSE118391). **(C, D)** Dual luciferase experiments indicating the fluorescence intensity of all five enhancers compared with that of the NC group in **(C)** AGS **(D)** MKN45 cells. ns, no significance; **p < 0.01, ***p < 0.001.

Since TM4SF1-AS1 was chosen as a representation of SE-associated lncRNAs in STAD, we further verified the regulation of the SE on TM4SF1-AS1. ChIP-seq data revealed one to two SE peaks in TM4SF1-AS1 in 11 STAD tumor samples and both AGS and MKN45 cell lines (**Figure 7A**). Subsequently, Hi-C data revealed that a spatial interaction exists between the SE regions and the TM4SF1-AS1 promoter (**Figure 7B**). We then cloned the five enhancer sequences and their negative control sequences into the PGL3-promotor vector for dual luciferase experiments. The fluorescence intensity of E2 and E3 was significantly higher than that of the NC group (**Figures 7C, D** **)**. We confirmed that TM4SF1-AS1, a SE-associated lncRNA, is indeed regulated by its super-enhancer in STAD based on the above results.

### TM4SF1-AS1 Is Involved in Immune Related Process

**Figure 8:**
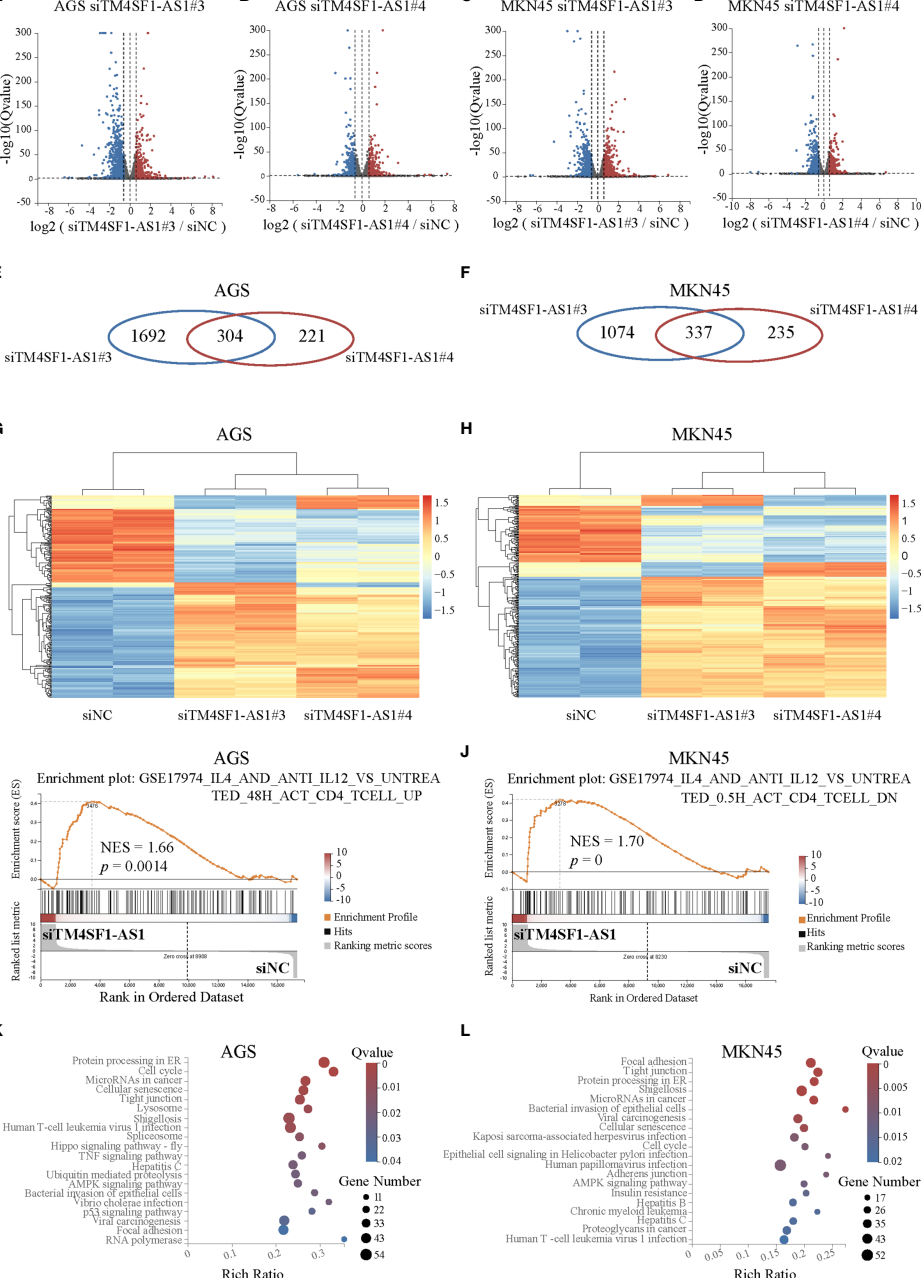
Pathway analysis of TM4SF1-AS1 knockdown in two STAD cells. **(A–D)** Volcano map showing the up- and down-regulated genes after TM4SF1-AS1 knockdown in AGS and MKN45 cells with two siRNA targets (siTM4SF1-AS1#3 and siTM4SF1-AS1#4). The red dots indicate up-regulated genes, and blue dots signify down-regulated genes. **(E, F)** Venn diagram displaying the differentially expressed genes by the intersection of siTM4SF1-AS1#3 and siTM4SF1-AS1#3 in **(E)** AGS and **(F)** MKN45 cells. **(G, H)** Heatmap showing the up- and down-regulated genes after TM4SF1-AS1 knockdown in **(G)** AGS and **(H)** MKN45 cells with siTM4SF1-AS1#3 and siTM4SF1-AS1#4. The red dots indicate up-regulated genes, and blue dots signify down-regulated genes. **(I, J)** GSEA analysis was performed on **(I)** AGS and **(J)** MKN45 cells following TM4SF1-AS1 knockdown. **(K, L)** KEGG analysis was performed on the changed genes in **(K)** AGS and **(L)** MKN45 cells after TM4SF1-AS1 knockdown with siRNAs. RNA-seq data of TM4SF1-AS1 knockdown in AGS MKN45 cells was from this paper.

Finally, we performed RNA-seq on AGS and MKN45 cells after treating them with TM4SF1-AS1 interference to further understand the tumor immune-related processes of TM4SF1-AS1 involved in STAD. First, we selected two siRNA targets, siTM4SF1-AS1#3 and siTM4SF1-AS1#4, for RNA-seq in two STAD cells. Next, we analyzed differentially expressed genes by comparing siTM4SF1-AS1 with siNC. We set the cut-off as log2|FC| ≥1 and Q<0.05, and identified both downregulated and upregulated genes following TM4SF1-AS1 knockdown in both AGS (**Figures 8A, B** **)** and MKN45 cells (**Figures 8C, D** **)**. The shared differentially expressed genes in groups between siTM4SF1-AS#3 and siTM4SF1-AS#4 of AGS and MKN45 cell lines were 304 and 337, respectively (**Figures 8E, F** **)**. Heatmap showed the expression change of shared differentially expressed genes in groups of siTM4SF1-AS#3, siTM4SF1-AS#4 in comparison with siNC group (**Figures 8G, H**). GSEA analysis revealed that TM4SF1-AS1 can regulate T cell-related biological processes in AGS (**Figures 8E, F** **)** and MKN45 cells (**Figures 8G, H** **)**. KEGG pathway analysis revealed that TM4SF1-AS1 modulates several biological processes and pathways, including protein processing in endoplasmic reticulum (ER), cell cycle, microRNAs in cancer, cellular senescence, and virus infection in AGS (**Figures 8I, J** **)** and MKN45 cells (**Figures 8K, L** **)**. These data further indicate that TM4SF1-AS1 is involved in tumor processes and immune regulation in STAD.

## Discussion

LncRNAs, which are transcribed by RNA polymerase II and independent transcriptional elements with lengths >200 bp, were thought to be unable to be translated into proteins (17). LncRNAs also play important roles in transcription, mRNA processing, post-transcriptional control, translation, and other biological processes (18, 19). Some lncRNAs are proven prospective markers of diagnosis, prognosis, and potential treatment targets in STAD, including EMT-associated lncRNA induced by TGFβ1 (ELIT-1) (20), LINC00346 (21), gastric cancer-associated lncRNA 1 (22) and gastric cancer metastasis-associated long noncoding RNA (23). LncRNAs play a crucial role in biological processes and have their own potential to act as biomarkers and therapeutic targets.

As mentioned before, lncRNAs can be epigenetically regulated by SEs to promote cancer malignancy. In ESCC, CCAT1 (24) and LINC01503 (25) are driven by SEs, which boost cancer progression. LncRNA UCA1 is activated by SE in cases of ovarian cancer, promoting tumor development (26). We previously found that SE-associated lncRNA HCCL5 was activated by ZEB1, which increased the malignancy of hepatocellular carcinoma (27). However, the biological function of SE-associated lncRNAs remains poorly characterized in tumors, especially in STAD. In the present study, we identified SE-associated lncRNAs in STAD for the first time to our knowledge and identified their potential oncogenic roles based on immune infiltration aspects and clinical outcomes.

The lncRNAs are reportedly involved in several biological processes containing the tumor immune microenvironment. Increased NKILA expression in T cells may aid cancer cells in escaping immunological destruction *via* activation-induced cell death (28). Low SATB2-AS1 expression enhances tumor metastasis and increases immune cell density in CRC (29). The roles of SE-associated lncRNAs in the tumor immune microenvironment of STAD where T cell immunity is present remains unknown. We first identified some SE-associated lncRNAs that were differentially expressed and correlated with immune cell infiltration in STAD in the present study.

Research on the tumor immune microenvironment has greatly promoted the development of tumor immunotherapy (30). T cells are the main cells involved in tumor immunotherapy and are responsible for defense against pathogen infection. It has been reported that TM4SF1-AS1 can exacerbate the progression of lung cancer (31), hepatocellular carcinoma (32) and gastric cancer (33). TM4SF1-AS1, a representative SE-associated lncRNA, was found to be involved in T cell immunity for the first time in the present study. Cancer cells evade the immune system’s screening and attack in several ways. Previous studies have confirmed that PD-1 and CTLA-4 are crucial for deactivating T cells (34, 35). PD-1, which is located on the membrane surface, interacts with PD-L1 and PD-L2 to exhaust effector T cells and reduce the immune response (35). In contrast, CTLA-4 is expressed in activated CD4+ and CD8+ T cells and interacts with the B7 molecule to inhibit T cell activation (36). Cancer patients currently benefit tremendously from both anti-PD1 and anti-CTLA-4 immunotherapies. In addition, some lncRNAs such as GATA3-AS1 (37), MIR17HG (38), UCA1 (39) and SNHG15 (40) regulate PD-L1 expression in several cancers. In the present study, SE-associated lncRNA TM4SF1-AS1 exhibited an immune response to anti-PD1 therapy, and was associated with the expression of PD1, PDL1, and PDL2.

In conclusion, we identified SE-associated lncRNAs in STAD samples and cells and found that they were correlated with the immune microenvironment and clinical prognosis of patients with STAD. As a representation of SE-associated lncRNAs, TM4SF1-AS1 participated in T cell immunity and monitors the immune response to anti-PD1 therapy. Therefore, some specific SE-associated lncRNAs can be used as potential targets to design specific drugs to regulate the tumor immune system and thereby kill tumors.

## Data Availability Statement

The original contributions presented in the study are included in the article/**Supplementary Material**. Further inquiries can be directed to the corresponding authors.

## Author Contributions

Conception and design: LP. Administrative support: LP, YL, X-QY and DY. Provision of study materials: LP and Q-SL. Collection and assembly of data: LP, J-YP, and D-KC. Data analysis and interpretation: LP, J-YP, X-QY, and YB. Writing and review of the manuscript: LP, Y-TQ, J-YP, JL, H-TX, Z-PD, and X-QY. All authors contributed to the article and approved the submitted version.

## Funding

This work was supported by the National Natural Science Foundation of China (grant no. 81972658 and 81802812 to LP), Guangdong Basic and Applied Basic Research Foundation (grant no. 2019A1515012114 and 2018A030313129 to LP), National Postdoctoral Program for Innovation Talents (grant no. BX20190395 to LP), China Postdoctoral Science Foundation (grant no. 2019M663254 to LP), the Fundamental Research Funds for the Central Universities (grant no. 20ykpy105 to LP). This study was also supported by grants from Guangdong Science and Technology Department (2020B1212060018, 2020B1212030004).

## Conflict of Interest

The authors declare that the research was conducted in the absence of any commercial or financial relationships that could be construed as a potential conflict of interest.

## Publisher’s Note

All claims expressed in this article are solely those of the authors and do not necessarily represent those of their affiliated organizations, or those of the publisher, the editors and the reviewers. Any product that may be evaluated in this article, or claim that may be made by its manufacturer, is not guaranteed or endorsed by the publisher.
