# Supplementary material for: Immune Infiltration and Clinical Outcome of Super-Enhancer-Associated lncRNAs in Stomach Adenocarcinoma
Source: Front Oncol. 2022 Mar 3;12:780493. doi: 10.3389/fonc.2022.780493 (PMC8927879; doi:10.3389/fonc.2022.780493)
Supplement: Supplementary file 1 [file DataSheet_1.docx]

Supplementary Material

## Supplementary Figures


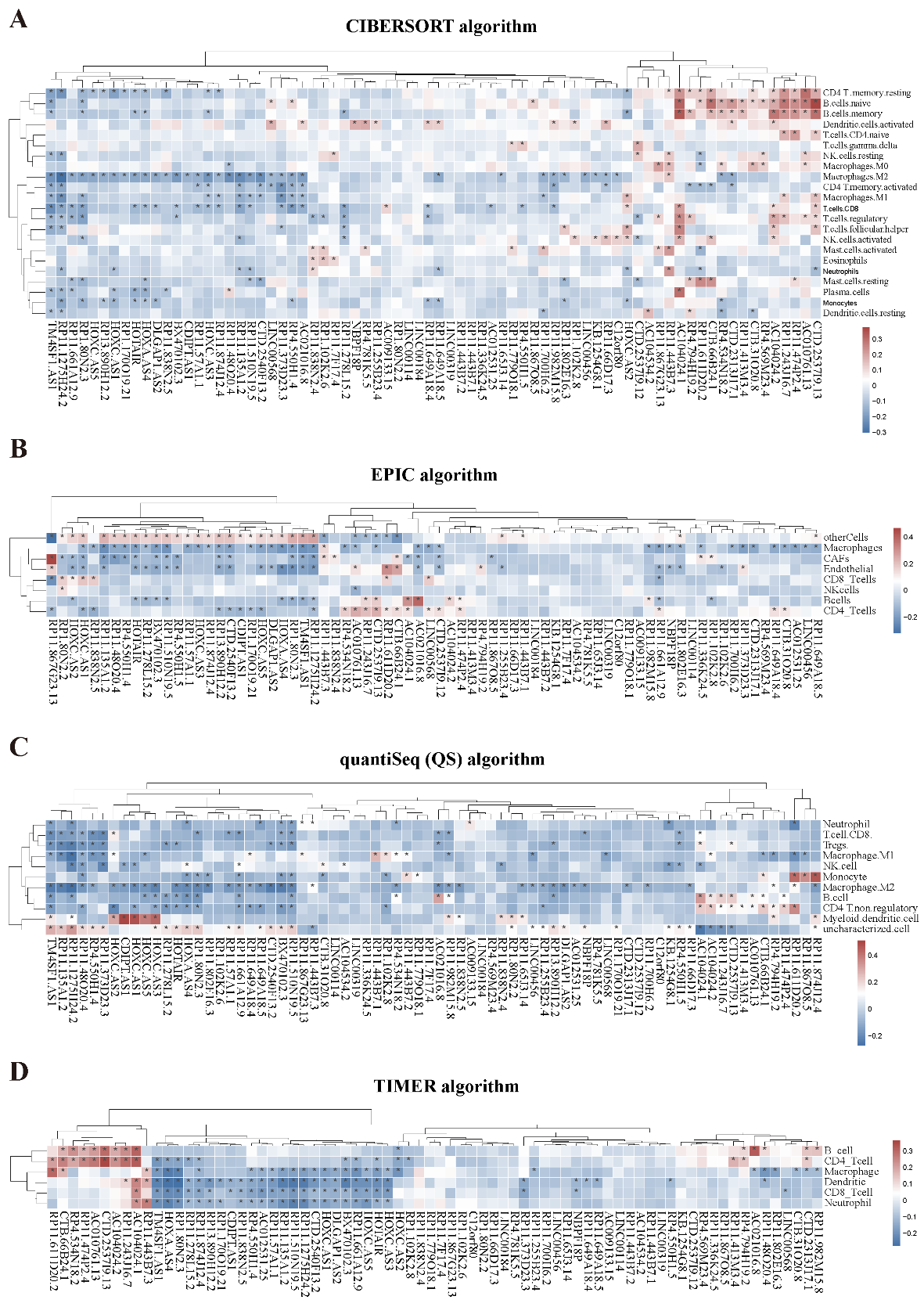


**Supplementary Figure S1.** The correlation between the expression of super-enhancer-associated lncRNAs and immune cell infiltration in STAD. (A-D) Cluster analysis of the correlation between 74 SE-associated lncRNAs expression and immune cell abundance calculated by using the (A) CIBERSORT, (B) EPIC, (C) quantiSeq (QS) and (D) TIMER algorithm. Red indicates positive correlation, while blue signifies negative correlation. The asterisk (*) indicates significant correlation (*p*<0.05).


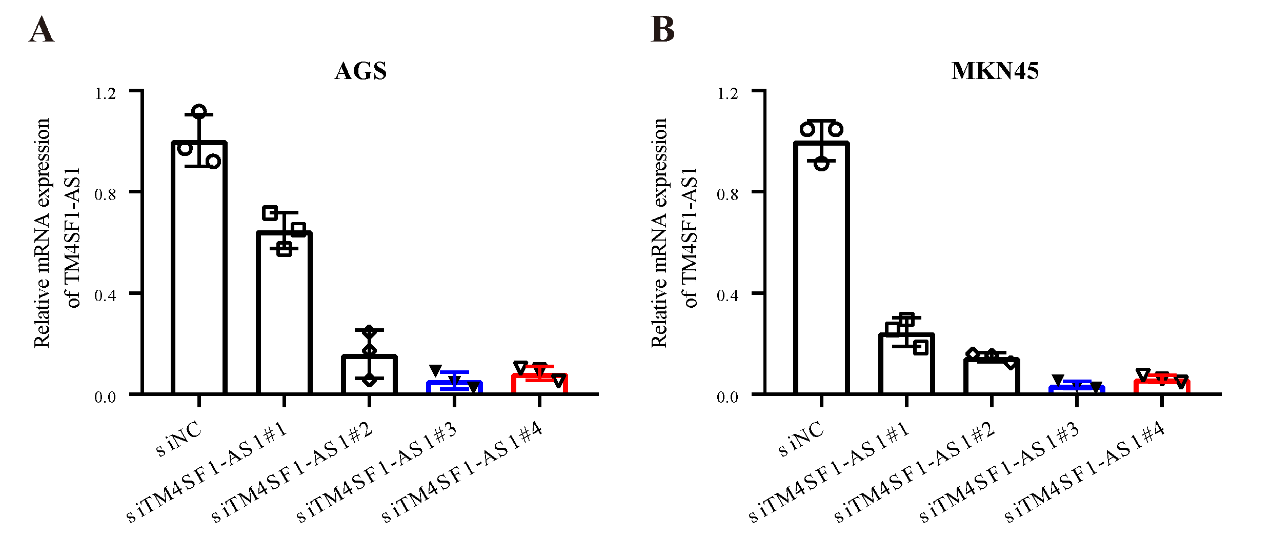


**Supplementary Figure S2.** Interference efficiency of TM4SF1-AS1 knockdown in two STAD cells. (A, B) The knockdown effect of four siRNA targets targeting TM4SF1-AS1 in (A) AGS and (B) MKN45 cell line.
